# A framework to identify gene expression profiles in a model of inflammation induced by lipopolysaccharide after treatment with thalidomide

**DOI:** 10.1186/1756-0500-5-292

**Published:** 2012-06-13

**Authors:** Renata T Paiva, Alessandra M Saliba, Tatiana O Fulco, Jorgenilce de Souza Sales, Daniel Serra de Carvalho, Elizabeth P Sampaio, Ulisses G Lopes, Euzenir N Sarno, Flavio F Nobre

**Affiliations:** 1 Biomedical Engineering Program, Federal University of Rio de Janeiro, Rio de Janeiro, RJ, Brazil; 2 Department of Microbiology, Immunology and Parasitology, State University of Rio de Janeiro, Rio de Janeiro, RJ, Brazil; 3 Leprosy Laboratory, Institute Oswaldo Cruz, Rio de Janeiro, Brazil; 4 Department of Molecular and Structural Biology, Federal University of Rio de Janeiro, Rio de Janeiro, RJ, Brazil

**Keywords:** Thalidomide, Microarray, Rank product, Inflammation model, Lipopolysaccharide

## Abstract

**Background:**

Thalidomide is an anti-inflammatory and anti-angiogenic drug currently used for the treatment of several diseases, including erythema nodosum leprosum, which occurs in patients with lepromatous leprosy. In this research, we use DNA microarray analysis to identify the impact of thalidomide on gene expression responses in human cells after lipopolysaccharide (LPS) stimulation. We employed a two-stage framework. Initially, we identified 1584 altered genes in response to LPS. Modulation of this set of genes was then analyzed in the LPS stimulated cells treated with thalidomide.

**Results:**

We identified 64 genes with altered expression induced by thalidomide using the rank product method. In addition, the lists of up-regulated and down-regulated genes were investigated by means of bioinformatics functional analysis, which allowed for the identification of biological processes affected by thalidomide. Confirmatory analysis was done in five of the identified genes using real time PCR.

**Conclusions:**

The results showed some genes that can further our understanding of the biological mechanisms in the action of thalidomide. Of the five genes evaluated with real time PCR, three were down regulated and two were up regulated confirming the initial results of the microarray analysis.

## Background

Thalidomide, which was widely marketed as a safe sedative and antiemetic for pregnant women between the years 1958 and 1960, was implicated in the birth of thousands of babies with congenital malformations in the following years. In 1965, the drug changed its course when it was showed to be effective in resolving symptoms associated with the inflammatory reaction of leprosy known as erythema nodosum leprosum, or type II reaction [1].

Currently, it is known that thalidomide is a drug with immunomodulating, anti-inflammatory and anti-angiogenic properties and that it is the drug of choice for the treatment of the type II leprosy reaction. Thalidomide is also used in some complications related to AIDS (acquired immunodeficiency syndrome), multiple myeloma, and chronic degenerative diseases, such as systemic lupus erythematosus and graft-versus-host disease [1-3]. Despite being approved for use in treating these diseases, and despite being used in experimental treatments of dozens of other diseases, such as heart failure and several types of solid tumors, the way that thalidomide mediates its anti-angiogenic and anti-inflammatory effects and the underlying molecular mechanisms involved has not been fully understood [4-6].

It has been demonstrated by several groups that thalidomide changes the levels of tumor necrosis factor (TNF), an important cytokine involved in many biological processes such as programmed cell death (apoptosis) and immune response [7-9]. It is also believed that the many biological activities associated with thalidomide can be explained largely by its effects on the activity of nuclear factor kappa B (NF-κB) [10]. NF-κB is a transcription factor involved in many physiological processes. These processes include the regulation of genes related to inflammation, such as cytokines *TNFIL6* and *IL2* proteins, the regulation of genes involved in apoptosis, such as baculoviral IAP repeat containing 3 (*BIRC3*) and family members’ *Bcl-2*, and in angiogenic factors, e.g., vascular endothelial growth factor (VEGF) [11].

The lipopolysaccharide (LPS) is a complex glycolipidic constituent of the outer cell wall of Gram-negative bacteria. LPS initiates its response through the stimulation of host cells such as monocytes and macrophages to produce and release endogenous mediators, including the pro-inflammatory cytokines IL-1, IL-6 and TNF [12]. MOREIRA et al. [13] examined the inhibitory action of thalidomide on TNF production induced by lipopolysaccharides and reported that the drug increases the degradation of TNF mRNA. The authors found that inhibition of TNF production was selective without changing other cytokines induced by LPS. The referred selective inhibition of TNF production allows the use of thalidomide for treating inflammatory conditions, where it is desirable to reduce TNF levels.

To further our understanding of thalidomide’s molecular mechanisms, we sought to explore the inflammation-suppressive effect of this drug on gene expression by using DNA microarrays in peripheral blood mononuclear cells (PBMC) stimulated with LPS as a model of inflammation.

Analysis of the molecular mechanisms of gene action can be achieved using DNA microarrays, which allows for the investigation of thousands of genes in a single experiment. Although advantageous, the use of microarrays presents some difficulties related to data analysis, including problems related to experimental noise, differences in dye incorporation and, in addition, the large number of genes to be statistically tested [14-17]. Different analytical approaches have been proposed to identify gene expression profiles, but no method has shown marked superiority over any other.

To identify genes associated with thalidomide treatment, we propose a two-stage procedure for microarray analysis. In the first stage, we use the rank product (RP) method of BREITLING et al. [18] on the residual term of the normalized LPS experiment with a less stringent statistical criteria to identify a list of genes responding to LPS stimulus. In the second stage, this set of genes was analyzed to identify a subset of expressed genes in the LPS stimulated cells treated with thalidomide.

## Results

### Microarray pre-processing

**Figure 1:**
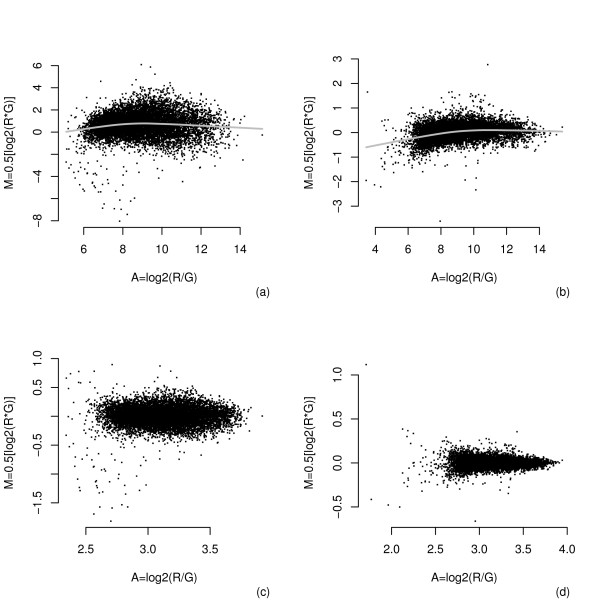
**MA-plots for one slide of each experimental condition.(a)** LPS and **(b)** LPS + Thalidomide before lowess transformation, **(c)** and **(d)** show the result of removing the intensity dependent curvature.

In most microarray experiments, the general assumption is that most of the genes will not be differentially expressed; therefore, the MA-plot should be evenly distributed around zero for all values of A. However, due to expected systematic biases in dye coupling, imbalances in hybridization efficiencies and other technical biases, the MA-plot may show the presence of these artifacts. The MA-plot is a plot of the Cy5 (R) versus Cy3 (G) obtained by plotting the intensity ratio $\text{M}={\text{log}}_{2}\left(\text{R}\right)–{\text{ log}}_{2}\left(\text{G}\right)$ by the average intensity $\text{A}=\left[{\text{log}}_{2}\left(\text{R}\right)+{\text{log}}_{2}\left(\text{G}\right)\right]/2$. The graph for the LPS experiment is depicted in Figure 1. The raw intensity data show a non-linear pattern in the MA-plot. Figure 1a and 1b show the raw data for one slide of each condition, LPS and LPS + thalidomide. For each slide, we applied the intensity global lowess regression approach for normalization and variability reduction of the data within the slides. The use of this transformation was efficient for properly removing the intensity dependent curvature present in the data, and the method scaled and centered each array (Figure 1c and 1d). Additional file 1: Figure A.1 and Additional file 2: Figure A.2 shows the MA plot for all arrays before and after lowess within normalization.

After the within normalization, we normalized the expression values to achieve consistency between arrays using the ANOVA model approach. The lowess centralized the data for the two channels in each array, but for further analysis to recognize differentially expressed genes, it is recommended that all arrays have a common reference level. This common reference level is obtained with the residual term *r* of the ANOVA model, which is the data used in the rank product method. See Additional file 3: Figure B.1 (a) Box plot after lowess transformation showing the centralization of the data for the two channels; (b) Box plot of the residual term of the ANOVA modeling.

The reproducibility of the arrays was evaluate by computing the Pearson correlation coefficient between the replicates within each array and between the probes among the arrays. The r-values for within array varies from 0.75 to 0.88 and between arrays from 0.25 to 0.65. See Additional file 4: Table A.1 shows the correlations for within arrays and table A.2 for the correlations between arrays.

### Expressed genes and their functional classes under LPS stimulation

After preprocessing the LPS arrays, the fold changes for the genes ranged from 0.016 to 9.25. The median fold change was 0.99, and about 50 % of the genes showed values twice or greater than the median value. Using the rank product statistics [18] we identify 1584 genes for further analysis. These genes were selected by using a nominal *p*-value threshold of 0.05. We did not control for multiple tests because we had only three arrays, which results in higher false discovery rates, and our main interest was in eliminating non-significant differentially expressed genes for the second stage of the analysis. Of the selected genes responding to LPS stimulus in our experiment, after a literature search, we found that 50 were also reported by SHARIF et al. [19], who analyzed the expression of NF-κB in macrophages in response to LPS using DNA microarrays of approximately 500 genes. Another six genes were reported by LEE et al. [20] who analyzed the expression profile of neutrophils obtained from skin biopsy material from patients with erythema nodosum leprosy. See additional file 5: Table B.1 shows the genes that were common to these two other studies focusing on LPS action.

To gain biological insights into the selected genes, a functional analysis was performed by searching for associations with Gene Ontology (GO) annotations to classify the genes altered by the LPS treatment into known functional processes. The gene universe was obtained by removing from the whole assayed genes those without GO representation, following the recommended procedure for using GOstats [21]. For the set of selected genes, the program finds the number of each GO term and counts the number of appearances of the term in the set of assayed genes in the experiment that have Entrez Gene identifiers or that map to available GO terms. A *p*-value for each GO term is computed indicating the probability that this term is randomly selected. Therefore, the GO terms with the lowest *p*-values are the ones most specific for the analyzed genes. Table 1 shows the biological process in differentially expressed genes in the LPS data obtained by selecting the GO categories with *p*-value < 0.05.

**Table 1:** Identification of LPS affected biological processes using the GOstats/R package for gene ontology analysis

**GOBPID**	**Pvalue**	**Count**	**Size**	**Term**
GO:0006414	0.000	36	78	translational elongation
GO:0042274	0.000	7	8	ribosomal small subunit biogenesis
GO:0042254	0.000	10	20	ribosome biogenesis
GO:0051591	0.001	6	9	response to cAMP
GO:0042416	0.004	3	3	dopamine biosynthetic process
GO:0050818	0.004	10	26	regulation of coagulation
GO:0015711	0.005	10	27	organic anion transport
GO:0032365	0.007	4	6	intracellular lipid transport
GO:0030195	0.007	8	20	negative regulation of blood coagulation
GO:0045730	0.011	5	10	respiratory burst
GO:0045022	0.013	3	4	early endosome to late endosome transport
GO:0014805	0.013	3	4	smooth muscle adaptation
GO:0032367	0.013	3	4	intracellular cholesterol transport
GO:0045410	0.013	3	4	positive regulation of interleukin-6 biosynthetic process
GO:0060325	0.013	3	4	face morphogenesis
GO:0030198	0.014	13	44	extracellular matrix organization
GO:0002293	0.014	4	7	alpha-beta T cell differentiation during immune response
GO:0042093	0.014	4	7	T-helper cell differentiation
GO:0045980	0.014	4	7	negative regulation of nucleotide metabolic process
GO:0006323	0.017	13	45	DNA packaging
GO:0030595	0.017	9	27	leukocyte chemotaxis
GO:0006333	0.017	14	50	chromatin assembly or disassembly
GO:0046635	0.018	5	11	positive regulation of alpha-beta T cell activation
GO:0045732	0.020	7	19	positive regulation of protein catabolic process
GO:0033993	0.024	4	8	response to lipid
GO:0002318	0.024	2	2	myeloid progenitor cell differentiation
GO:0002523	0.024	2	2	leukocyte migration during inflammatory response
GO:0008588	0.024	2	2	release of cytoplasmic sequestered NF-kappaB
GO:0015808	0.024	2	2	L-alanine transport
GO:0015824	0.024	2	2	proline transport
GO:0018347	0.024	2	2	protein amino acid farnesylation
GO:0019614	0.024	2	2	catechol catabolic process
GO:0031440	0.024	2	2	regulation of mRNA 3'-end processing
GO:0032417	0.024	2	2	positive regulation of sodium:hydrogen antiporter activity
GO:0032510	0.024	2	2	endosome to lysosome transport via multivesicular body sorting pathway
GO:0033625	0.024	2	2	positive regulation of integrin activation
GO:0033630	0.024	2	2	positive regulation of cell adhesion mediated by integrin
GO:0034109	0.024	2	2	homotypic cell-cell adhesion
GO:0042420	0.024	2	2	dopamine catabolic process
GO:0042832	0.024	2	2	defense response to protozoan
GO:0043179	0.024	2	2	rhythmic excitation
GO:0045627	0.024	2	2	positive regulation of T-helper 1 cell differentiation
GO:0045628	0.024	2	2	regulation of T-helper 2 cell differentiation
GO:0048103	0.024	2	2	somatic stem cell division
GO:0048539	0.024	2	2	bone marrow development

The analyses showed the biological process regulation of important classes such as the regulation of T-helper cell differentiation, the negative regulation of blood coagulation, the regulation of coagulation, alpha-beta T cell activation involved in immune responses, T cell differentiation involved in immune responses, CD4-positive, alpha-beta T cell differentiation involved in immune responses, cell chemotaxis, and positive regulation of receptor-mediated endocytosis terms that are related to an inflammatory/immune response induced by LPS. Besides these, other terms were also found altered by LPS, such as smooth muscle adaptation nerve development, intracellular cholesterol transport, and intracellular lipid transport.

Analysis of cellular components (parts of the cell or extra cellular environment) revealed several genes in the cytosol component (GO: 0005829), with 158 expressed genes, where 82 genes are over-expressed. Another cellular component that showed the greatest number of genes was non-membrane-enclosed organelles (GO: 0043228), with 255 genes represented in this set, including 145 over-expressed genes. In the category molecular function (the elemental activities of the gene product at the molecular level), the over-represented profiles corresponded to categories of GTPase regulator activity (GO: 0030695), with a total of 41 genes in the profile, where 25 are over-expressed, and there was enzyme regulator activity with 88 genes in the profile (52 genes over-expressed).

While GO annotations can summarize and simplify the interpretation of the selected genes, additional enrichment analysis for these genes can be gained by identifying metabolic pathways in the Kyoto Encyclopedia of Genes and Genomes (KEGG). For this analysis, we also used the GOstats package, and the cut-off was *p* < 0.05 as well. Table 2 shows the enriched metabolic pathways identified from the KEGG database. From this analysis, for the over-represented KEGG pathways, it is possible to observe several genes identified in the ribosome pathway (hsa03010) and the glutathione metabolism pathway (hsa00480), with 32 genes and 10 genes, respectively, in addition to fructose and mannose metabolism pathways, DNA replication, phototransduction, and ABC transporters.

**Table 2:** Identification of biological pathways via KEGG for LPS

**KEGGID**	**Pvalue**	**Count**	**Size**	**Term**
3010	0.000	30	62	Ribosome
51	0.018	9	27	Fructose and mannose metabolism
3030	0.032	8	25	DNA replication
480	0.038	10	35	Glutathione metabolism
2010	0.040	8	26	ABC transporters

The analysis of LPS activated cultures led to the identification of genes involved in processes or pathways related to inflammation. This provides a more specific analytic set of genes for studying the mechanism of action of thalidomide in inflammation because only those genes related to inflammation are considered.

### Modulation of LPS induced genes by thalidomide

Only genes selected in the LPS experiment were taken into account for further analysis involving the identification of genes that were altered when treated with thalidomide. In this case, the gene expression values were again log-transformed before preprocessing with lowess and ANOVA following a similar approach as described for the LPS data set. After these preprocessing stages, the LPS + thalidomide residual data had a range of fold changes for the three arrays varying from 0.016 to 9.25. Rank product analysis was used to obtain a subset of 64 genes altered by the action of thalidomide (Table 3) using a cutoff nominal *p*-value of 0.001. Again, we did not considered multiple test correction due to the small sample size. Here, we used a more stringent cutoff level because we are more interested in reducing the occurrence of type I error (false positive) due to the possibility of coordinated expression of genes in the inflammation model. For the selection of genes related to the inflammation process, the presence of false positive genes was not critical. The importance here was to avoid the presence of genes with coordinated expression, since the RP method assumes the independence of genes and may lead to underestimation when the values of the expression of many genes at the top of the list are dependent.

**Table 3:** Genes with altered expression due to thalidomide action

**Down regulated genes**				**Up-regulated genes**			
**Acc. Number**	**Symbol**	**FC**	**P value**	**Acc. Number**	**Symbol**	**FC**	**P value**
NM_022052	NXF3	0.39	0.0000	XM_030485		2.98	0.0000
NM_003192	TBCC	0.53	0.0000	NM_017660	p66alpha	2.91	0.0000
NM_020346	SLC17A6	0.54	0.0001	NM_000994	RPL32	2.18	0.0000
NM_000120	EPHX1	0.62	0.0002	NM_002032	FTH1	2.25	0.0000
NM_004081	DAZ4	0.62	0.0002	NM_001028	RPS25	2.16	0.0000
NM_002069	GNAI1	0.63	0.0003	NM_033496	HK1	2.21	0.0001
NM_022066	E2-230 K	0.53	0.0003	AF245436	FLJ23518	2.10	0.0001
NM_006468	POLR3C	0.63	0.0005	NM_024552	LASS4	2.01	0.0002
NM_018230	NUP133	0.56	0.0006	NM_023071	SPATS2	1.98	0.0004
NM_000303	PMM2	0.62	0.0007	NM_015937	PIGT	1.95	0.0004
NM_001868	CPA1	0.65	0.0008	NM_005896	IDH1	1.69	0.0005
NM_022163	MRPL46	0.63	0.0008	NM_006995	BTN2A2	1.68	0.0006
AF113016	PRO1073	0.57	0.0008	NM_003690	PRKRA	1.79	0.0009
NM_017708	FLJ20200	0.62	0.0009	NM_016025	DREV1	2.06	0.0009
NM_021737	CLCN6	0.66	0.0009	NM_021734	SLC25A19	1.75	0.0009
NM_002852	PTX3	0.69	0.0011	NM_022663	CTAGE1	1.76	0.0009
NM_000042	APOH	0.67	0.0011	NM_019848	SLC10A3	1.90	0.0010
NM_006052	DSCR3	0.67	0.0014	NM_022126	LHPP	1.71	0.0011
XM_007829		0.68	0.0017	NM_001493	GDI1	1.69	0.0012
NM_002630	PGC	0.67	0.0018	BC003599	BM-009	1.57	0.0013
NM_003033	SIAT4A	0.68	0.0019	NM_001825	CKMT2	1.83	0.0014
NM_001146	ANGPT1	0.68	0.0020	NM_013243	SCG3	1.83	0.0017
NM_014872	ZBTB5	0.69	0.0022	NM_005505	SCARB1	1.59	0.0018
NM_002819	PTBP1	0.66	0.0023	NM_024841	FLJ14213	1.61	0.0019
NM_024076	KCTD15	0.70	0.0024	NM_005557	KRT16	1.55	0.0022
NM_001586	CXorf2	0.68	0.0025	NM_003205	TCF12	1.56	0.0025
NM_017777	FLJ20345	0.70	0.0025	NM_003652	CPZ	1.58	0.0026
NM_003626	PPFIA1	0.71	0.0028	NM_001012	RPS8	1.55	0.0026
NM_006858	IL1RL1LG	0.70	0.0032	NM_001003	RPLP1	1.51	0.0027
NM_033540	MFN1	0.71	0.0046	NM_004576	PPP2R2B	1.56	0.0027
				NM_004202	TMSB4Y	1.55	0.0030
				NM_001626	AKT2	1.50	0.0030
				NM_001152	SLC25A5	1.57	0.0030
				NM_017452	STAU	1.59	0.0037
				NM_000576	IL1B	1.59	0.0049

The table shows the accession number (Acc. number), the gene symbol, the fold-change (FC) and the Pvalue.

We examined whether the selected genes under a thalidomide treatment share similar biological functions or are within similar functional classes and pathways of the LPS experiment using GOstats. Analysis of the association between gene and class in the category of biological process gene ontology identified terms that were more significant within the group (Table 4). The association analysis using the gene-class KEGG also identified pathways that may be changed by thalidomide (Table 5).

**Table 4:** Identification of biological processes using gene ontology affected by the action of thalidomide

**GOBPID**	**Pvalue**	**Count**	**Size**	**Term**
GO:0015931	0.006	3	9	nucleobase, nucleoside, nucleotide and nucleic acid transport
GO:0045429	0.011	2	4	positive regulation of nitric oxide biosynthetic process
GO:0051168	0.011	2	4	nuclear export
GO:0044267	0.017	18	246	cellular protein metabolic process
GO:0051707	0.022	3	14	response to other organism
GO:0046209	0.027	2	6	nitric oxide metabolic process
GO:0048514	0.032	3	16	blood vessel morphogenesis

**Table 5:** KEEG enrichment and thalidomide

**KEGGID**	**Pvalue**	**Count**	**Size**	**Term**
4530	0.011	3	11	Tight junction
533	0.046	1	1	Keratan sulfate biosynthesis
604	0.046	1	1	Glycosphingolipid biosynthesis - ganglio series
1031	0.047	2	8	Glycan structures - biosynthesis 2
4620	0.047	2	8	Toll-like receptor signaling pathway
51	0.059	2	9	Fructose and mannose metabolism
4210	0.059	2	9	Apoptosis
3010	0.064	3	21	Ribosome
52	0.089	1	2	Galactose metabolism
512	0.089	1	2	O-Glycan biosynthesis
720	0.089	1	2	Reductive carboxylate cycle (CO2 fixation)
5213	0.089	1	2	Endometrial cancer
5218	0.089	1	2	Melanoma
5223	0.089	1	2	Non-small cell lung cancer

### Evaluation of gene expression by RT-PCR

**Figure 2:**
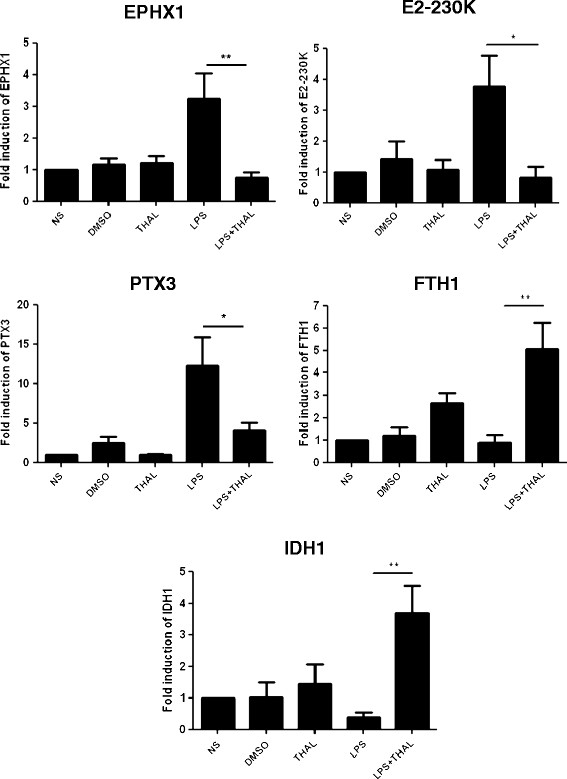
**Thalidomide modulates gene expression induced by LPS in vitro.** PBMC obtained from healthy donors were pre-treated with thalidomide (30 min) and stimulated or not (NS) with LPS (1μg/mL) for 3 h. As an additional control, non-stimulated cells were also maintained in culture with DMSO alone (0.1 %). Results are mean ± SEM of four independent experiments carried out in duplicate. Expression of genes induced by LPS, EPHX1, E2-230 K and PTX3 were down regulated In the presence of thalidomide whereas FTH1 and IDH1 were upregulated. Statistical differences between groups were determined via a one-way analysis of variance (ANOVA) followed by the Bonferroni’s multiple comparison post-test: *p < 0.05; **p < 0.01 indicate significant differences when compared to cultures stimulated with LPS alone.

For these experiments, PBMC from 4 healthy donors were cultured and stimulated as described above. RNA was isolated from the cultures, reverse transcribed and expression of five genes was assayed by real time PCR. Three of them (*EPHX1, UBE2O, PTX3*) were down regulated (4–5 fold reduction) in cells treated with thalidomide whereas other two genes, *FTH1* and *IDH1*, were up regulated (Figure 2) when compared to cultures stimulated with LPS alone. These data are in agreement with the microarray analysis.

## Discussion

Despite the recognized clinical merits of thalidomide, the molecular mechanism of the action of this drug still remains unclear. Over the past 10 years, even in the face of increasing development of microarray technology to study gene expression profiles, there is a limited amount of microarray data related to thalidomide, and most studies focus on the use of this drug in different types of cancers [22-24]. Therefore, this study was undertaken to understand the mechanism of action of thalidomide in the inhibition of inflammatory processes.

In this paper, we describe a two-stage framework. In the first stage, we identified a set of differentially expressed genes in a model of inflammation induced by lipopolysaccharide. The analysis of this experiment identified 1584 genes that may be altered by the inflammatory process. We used a cutoff nominal *p*-value of 0.05 during this first stage to avoid possible missing genes that, even with low fold changes, may be induced by the action of thalidomide. Therefore, we increase the chance to have larger numbers of false positives in the subset of selected genes during this first stage since in the second stage of analysis, these genes could be relevant to drug action. In the second stage, the analysis of the experiment with thalidomide identified 64 differentially expressed genes, among which 30 were under-expressed genes and 34 over-expressed genes.

In this study, we used the rank product method as a basic tool for identifying differentially expressed genes. This method is based on the ratio of expression, where mRNA levels are compared under different conditions (A and B) on a slide. It is an approach that allows for an easier interpretation of the results. Use of the rank product method has been recommended after appropriate normalization for situations where there are few replicates [25]. The raw data showed systematic deviations that were removed using normalization techniques. Using the logarithmic transformation, the ratios between the two channels are expressed in terms of differences, and it stabilizes the variance of the wells of high intensity. After the logarithmic transformation, the data showed characteristic intensity dependence and, as a result, were normalized using the lowess method. This method of local regression curve fitting reduced the variability dependent on the intensity, allowing biological differences to be better visualized. Finally, the ANOVA provided a method to normalize between arrays and the residue from this fitting is used as the data for the rank product method.

The list of genes identified as differentially expressed in the experiment with thalidomide can provide information about the mechanism of action of this drug. However, obtaining this list is not enough to provide information related to the biological mechanisms involved on these altered profiles. To help the interpretation of the data, we apply techniques of gene ontology analysis that are freely available to categorize the differentially expressed genes according to functional processes. We also used pathway analysis for the identification of metabolic pathways altered, providing further information regarding the mechanisms of action of thalidomide.

The enrichment analysis allowed the identification of biological processes affected by thalidomide. Among these processes, we identified the morphogenesis of blood vessels that may explain the anti-angiogenic properties of thalidomide, with angiopoietin 1 (*ANGPT1*) identified as under-expressed. The protein encoded by this gene is a secreted glycoprotein that activates the receptor, inducing its phosphorylation. This protein plays a key role in mediating reciprocal interactions between the endothelium and the underlying matrix as well as in the mesenchyme. The protein also contributes to the stabilization and maturation of blood vessels and may be involved in the initial development of the heart [26]. In addition, other genes were identified that are involved in apoptosis mechanisms and in signaling pathways, such as the polypeptide protein tyrosine phosphatase, the receptor type F (*PTPRF*), a member of the protein phosphatase family that regulate a variety of cellular processes including cell growth, differentiation, mitotic cycle, and oncogenic transformation [27,28]; the interferon inducible double stranded RNA dependent activator (*PRKRA*), which encodes a protein kinase that mediates the effects of interferon in response to viral infection [29].

Among the genes validated by RT-PCR, *UBE2O* is an E2 ubiquitin ligase, it has a role in regulating protein degradation [30]. Pentraxin-3, *PTX3*, belongs to the acute phase proteins family produced mainly by macrophages, dendritic cells and endothelial cells in response Toll-like receptor ligands and inflammatory cytokines (eg. TNF and IL-1β) [31]; Ferritin, heavy polypeptide 1 (*FTH1*) is a subunit of the most important iron storage protein, plays an essential role in iron homeostasis and a wide range of physiologic processes. Recently it was described the interaction of FTH1 to the pathway *Fas-Daxx-ASK1-JNK1*, demonstrating its properties to inhibit apoptosis mediated by *Daxx* and inhibition of *JNK* activation [32]. The raise of *FTH1* gene expression by thalidomide can also imply in the immunomodulation activity of this drug due to possible *FTH1* inhibition of *JNK* activation. *EPHX1* encodes the gene of epoxide hydrolase 1, it biotransforms epoxide derivatives of pharmaceuticals, including metabolites of certain antiepileptic medications [33].

Thalidomide had the ability to induce *IDH1* gene expression, Isocitrate dehydrogenase (*IDH*)-1 mutations are associated to glioma development. *IDH1* mutations in arginine 132 result in a new ability of the enzyme to catalyse the NADPH-dependent reduction of alpha-ketoglutarate to R(−)-2-hydroxyglutarate (2HG) [34].

The biological processes of the genes altered by the LPS inflammation model, which were affected by thalidomide, are processes related to angiogenesis and to the response to metabolic stress; a microorganism could for example, trigger these processes. No direct action of thalidomide on the gene expression of inflammatory cytokines was observed, but thalidomide’s action occurs through precursor processes of the inflammation. A similar result was recently reported by NOMAN et al. [35] in a study of the inhibition of TNF production in RAW 264.7 cells. In that work, the authors show that thalidomide has an indirect effect on LPS signaling via down-regulation of the MyD88 protein. This universal adapter protein is used by all Toll-Like Receptors (except *TLR3*) to activate the transcription factor NF- κB and mRNA and inhibits LPS-induced TNF production.

## Conclusions

In conclusion, we carried out a two-stage procedure of microarray analysis to detect genes associated with thalidomide treatment under an inflammation model. The proposed method detected effects that may fail to emerge using a single experiment with LPS and thalidomide. To find statistically significant changes in gene expression with our small data set, we used the rank product method, which is a flexible technique for detecting differentially expressed genes, even under adverse conditions such as with restricted replications [25]. Of the 64 identified genes, five were evaluated using real time PCR to provide more consistent evidence of their identified gene expression profiles.

## Methods

### Reagents

Lipopolysacharide (LPS) from Salmonella Minnesota Re 595 was purchased from Sigma Chemical Co. (St. Louis, MO). Thalidomide was obtained from Calbiochem (Cambridge, MA); DMSO (Sigma) was used as a solvent for thalidomide and the final concentration of DMSO in the cell cultures was 0.1 %.

### Cell culture and stimulation

Buffy coats were obtained from three healthy donors at the Hemotherapy Service of the Clementino Fraga Filho University Hospital, associated with the Federal University of Rio de Janeiro, RJ, Brazil, in accordance with the guidelines set down in the Declaration of Helsinki. The acquisition of all specimens was approved by the Human Ethics Committee of the Oswaldo Cruz Foundation, Brazil.

Isolation of peripheral blood mononuclear cells (PBMC) was performed under endotoxin free conditions through Ficoll-Hypaque (Pharmacia Fine Chemicals, Piscataway, NJ) density centrifugation. Cells (5x106/well) were cultured in 6-well plates (Costar Corporation, Cambridge, MA), in RPMI 1640 medium supplemented with 100U/ml penicillin, 100 μg/ml streptomycin, 2 mM L-glutamine, and 10 % FCS (Gibco BRL, Gaithersburg, MD) at 37 °C. Cultures were pre-treated or not with thalidomide (25 μg/ml) or were left unstimulated or were stimulated with LPS (1 μg/ml) for an additional 3 hour. After the stimulation period, cells were recovered and lysed for RNA isolation by using tryzol (Gibco, BRL), processed according to the manufacturer’s recommendation. The experiments considered a pool of RNAs obtained for each experimental condition (non-stimulated untreated cells; cells stimulated with LPS alone; cells treated with thalidomide alone; cells treated with LPS and thalidomide).

### Microarray

The microarray data were obtained through a partnership between the Institute of Biophysics Carlos Chagas Filho (UFRJ), the Leprosy Laboratory, FIOCRUZ and the DNA Microarray Facility at the Universidad Nacional Autónoma de México (UNAM - Mexico). Oligonucleotides (50 mer) representing 9984 human genes, purchased by UNAM from the MWG Biotech Company (http://www.mwg-biotech.com), were spotted in duplicate onto superamine-coated glass slides (Telechem International) using the Virtek ChipWriter, resulting in a total of 19,968 spots. For each treatment condition, when we compared LPS, thalidomide or LPS + thalidomide, target cDNA was hybridized to three microarrays. To reduce the bias of intensity associated with the difference in dye incorporation, for two arrays, the stimulated samples (LPS, thalidomide or LPS + thalidomide) were labeled using red-fluorescent dye, Cy5, and the non-exposed samples were assigned to the green-fluorescent dye, Cy3. For the third array, dye labeling was reversed. The experimental microarray design used here is known as “dye swap.” After standard pre-hybridization for 1 h at 42^0^ C, samples taken from the untreated cells (control) and treated cells were added to buffer hybridization Hybit 2 (Telechea International), and they were hybridized overnight in each array at 42^0^ C in a humidified chamber (Corning). The hybridized slides were then washed, dried and scanned using the Scan Array 4000 (Packard Biochips).

The intensities of the spots for each array were obtained with the Array Pro Analyzer software, which generates two 16 bit images, one for each dye. For each array, the values of the gene expression were obtained by subtracting for each spot its averaged background from the mean foreground value. For cases with resulting values less than or equal to zero, we replaced them with the internal replicate if these were different from zero; otherwise, the gene was eliminated. After background correction, the mean values of the replicates were used to obtain the logarithm with base two, resulting in the expression level *y*_*ijkg*_ for each array. Initial analysis involved the production of MA-plots for the arrays (Figure 1).

This exploratory tool is useful to identify systematic variations due to different dye labeling efficiencies, differences in the concentration of DNA on arrays, and other effects. The MA-plot reveals whether the data exhibit an intensity-dependent structure. The presence of systematic effects impairs a proper analysis of individual slides. Comparison of expression levels between slides show a non-linear pattern in the MA-plot, indicating the use of data normalization to remove these effects. Here, we used lowess, a locally weighted scatter plot smoothing for within slide normalization and variability reduction [36].

The use of this transformation is efficient to properly scale and to center the data for each slide. For proper comparison between arrays, it is still necessary to center all arrays at zero, and this task has been achieved with the use of the ANOVA model [37]. The ANOVA model was used only for normalization between slides. The model considered the sources of variability that are usually present in a microarray experiment, and is defined for each gene *g* as:

(1) $$y_{\mathrm{ijkg}}=\mu +A_{i}+D_{j}+AD_{\mathrm{ij}}+r_{\mathrm{ijkg}}$$

where *A* and *D* are the array *i*, and the dye effects, *j. AD* is the interaction between dye and array and *μ* captures the array global mean for all genes, *g*, and the experimental condition is *k*. The *r* term accounts for unexplained factors and is assumed as the error term with zero mean. This residue from the ANOVA model was used as the main data for statistical analysis using the non-parametric rank product statistics [18] to identify differentially expressed genes.

The entire normalization process, using the lowess transformation and normalization with the ANOVA model, was done with the package MAANOVA/R [38]. This preprocessing stage reduced or eliminated the variability introduced by differences in the efficiency of dye incorporation and the differences in experimental conditions that may influence the intensity of hybridization. To carry out the rank product analysis, we used the Bioconductor R/Rank Prod package [39].

The rank product (RP) approach was selected because it is a nonparametric test and does not require stringent assumptions of probability distributions. It is also a method that is less prone to be influenced by experimental noise, being applicable to experiments with a small number of arrays [25], as in our case, in which there were only three arrays for each experimental condition. The RP method is based on rank ordering the fold change of the genes for each array, followed by computing a geometric mean of the ranks. Converting the fold changes into ranks provides a more robust measure across arrays even with a poor correlation across platforms. Genes associated with the smallest ranks are the ones that should be marked for further consideration. The RP computed values is evaluated against a sampling distribution obtained using permutation to generate nominal *p*-values and also false discovery rates. A list of up- or down- expressed genes can be obtained using a chosen threshold level. Here we use the nominal *p*-values for the ranked genes.

To further our understanding of the thalidomide mechanism of action, as a first step, we considered the LPS infection model and we identified a list of up- or down regulated genes using a less stringent *p*-value so that a larger number of genes would be selected as altered by the LPS inflammation effect. The lists of selected differentially expressed genes in response to LPS were submitted to a functional analysis based on its association to Gene Ontology (GO) terms. This enrichment analysis allowed an integrated information of biological processes altered by LPS. Association between the genes and their classes, as defined in the databases of Gene Ontology (Gene Ontology) and KEGG pathways (Encyclopedia of Genes and Genomes Kyoto), was evaluated using the hypergeometric test available in GOstats [21], a Bioconductor package written in R. Additionally, we did a literature search to identify reported altered genes under LPS stimulus that are common to our selected set.

The set of genes selected in this first stage of analysis was then investigated in the LPS + thalidomide experiments. It is worth noting that all methods used for the LPS experiment were also applied for the identification of differentially expressed genes within this experiment.

### Real time PCR

For validation of the microarray results, PBMC obtained from 4 healthy donors were cultured and stimulated under the same conditions as described above. Cells were pre-treated or not with thalidomide (30 min), and stimulated with LPS 1ug/ml for 3 h, when total RNA was extracted by using Trizol. For RT-PCR, 1μg of total RNA was reverse transcribed using oligo-dT primers (Life Technologies) and the resulting cDNA was amplified by Real time RT-PCR using the ABI 7500 Sequencer and the Taqman expression assays (Applied Biosystems). For detection of genes, primers used were: *EPHX1 Hs01116806_m1, UBE2O Hs00222904_m1, PTX3 Hs00173615_m1, FTH1 Hs01694011_s1, IDH1 Hs00271858_m1*. *GAPDH* was used as normalization control. The data were analyzed using the 2^-ΔΔ*C*^_T_ method.

### Availability of supporting data

The data sets supporting the results of this article are available at GEO repository in http://www.ncbi.nlm.nih.gov/geo/query/acc.cgi?token=pzafdaawusimezk&acc=GSE28708.

## Competing interest

The authors declare that they have no competing interests.

## Authors' contributions

RTP define and carried out the statistical, computational analysis, interpretation of the results and wrote most of the paper. FFN contribute in the definition of the computational methods to be used and in the writing of the paper. ENS and UGL conceived the study and the design protocol. AMS, TOF and EPS participate in the design of the study and coordination of the experiments and data acquisition. JSS and DSC did the RT-PCR analysis. All authors read and approved the final manuscript.

## Supplementary Material

Additional file 1: Figure A1 MA plot of all arrays before normalization with lowess. Click here for file

Additional file 2: Figure A2 MA plot of all arrays after lowess normalization. Click here for file

Additional file 3: Figure B1 (a) Box plot after lowess transformation showing the centralization of the data for the two channels; (b) Box plot of the residual term of the ANOVA modeling. A1 to A6, LPS arrays; A7 to A12, LPS + Thalidomide arrays and A13 to A18, thalidomide arrays. Click here for file

Additional file 4: Table A.1_2 Table A.1 Correlation between the arrays for the three experimental conditions and Table A.2 Correlation within arrays for the three experimental conditions. Click here for file

Additional file 5: Table B1 Genes identified as changed by LPS, as reported in this work, and those of SHARIF et al. [19] and LEE et al. [20]. Click here for file
