# Supplementary material for: A framework to identify gene expression profiles in a model of inflammation induced by lipopolysaccharide after treatment with thalidomide
Source: BMC Res Notes. 2012 Jun 13;5:292. doi: 10.1186/1756-0500-5-292 (PMC3434117; doi:10.1186/1756-0500-5-292)
Supplement: Additional file 1: Figure A1 — MA plot of all arrays before normalization with lowess. [file 1756-0500-5-292-S1.pdf]

Table A.1 Correlation between the arrays for the three experimental conditions.

	Array1 vs Array2	Array1 vs Array3	Array2 vs Array3
LPS	0.65	0.65	0.51
LPS+Thal	0.25	0.52	0.41
Thal	0.28	0.58	0.54

Table A.2 Correlation within arrays for the three experimental conditions

	Array1	Array2	Array3
LPS	0.88	0.75	0.80
LPS+Thal	0.87	0.83	0.78
Thal	0.81	0.75	0.83
