# Supplementary material for: A framework to identify gene expression profiles in a model of inflammation induced by lipopolysaccharide after treatment with thalidomide
Source: BMC Res Notes. 2012 Jun 13;5:292. doi: 10.1186/1756-0500-5-292 (PMC3434117; doi:10.1186/1756-0500-5-292)
Supplement: Additional file 2: Figure A2 — MA plot of all arrays after lowess normalization. [file 1756-0500-5-292-S2.pdf]

Table B.1: Genes identified as changed by LPS, as reported in this work, and those of SHARIF et al. [19] and LEE et al. [20].

Autor	Genes					
SHARIF et al (2007)	CCL24	HAS3	GDF15	TNFSF13B	TP53	EPOR	SLC3A2
	MST1R	SFPQ	APP	CSF1R	C4A	FLT3LG	HMOX1
	TNFSF5	TNFSF15	AGT	KRT19	KRT19	ICAM3	HMOX1
	IL1R2	FCER2	BTG2	GAD1	TNFSF8	TNFRSF21	TAP2
	IFNGR1	PAEP	SELP	SELE	IFNAR1	BTG2	TCERG1
	AMPD2	TGFA	IL9	HAS2	BLR1	NDRG1	HAS2
	HMGB1	DSC2	MMP9	TAP2	IL13RA1	HMMR	GDF15
	S100A1	ICAM3	MMP9				
LEE et al (2010)	ITGA5	IL2RA	SELP	SELE	B4GALT1		
