# Supplementary material for: A framework to identify gene expression profiles in a model of inflammation induced by lipopolysaccharide after treatment with thalidomide
Source: BMC Res Notes. 2012 Jun 13;5:292. doi: 10.1186/1756-0500-5-292 (PMC3434117; doi:10.1186/1756-0500-5-292)
Supplement: Additional file 3: Figure B1 — (a) Box plot after lowess transformation showing the centralization of the data for the two channels; (b) Box plot of the residual term of the ANOVA modeling. A1 to A6, LPS arrays; A7 to A12, LPS + Thalidomide arrays and A13 to A18, thalidomide arrays. [file 1756-0500-5-292-S3.pdf]

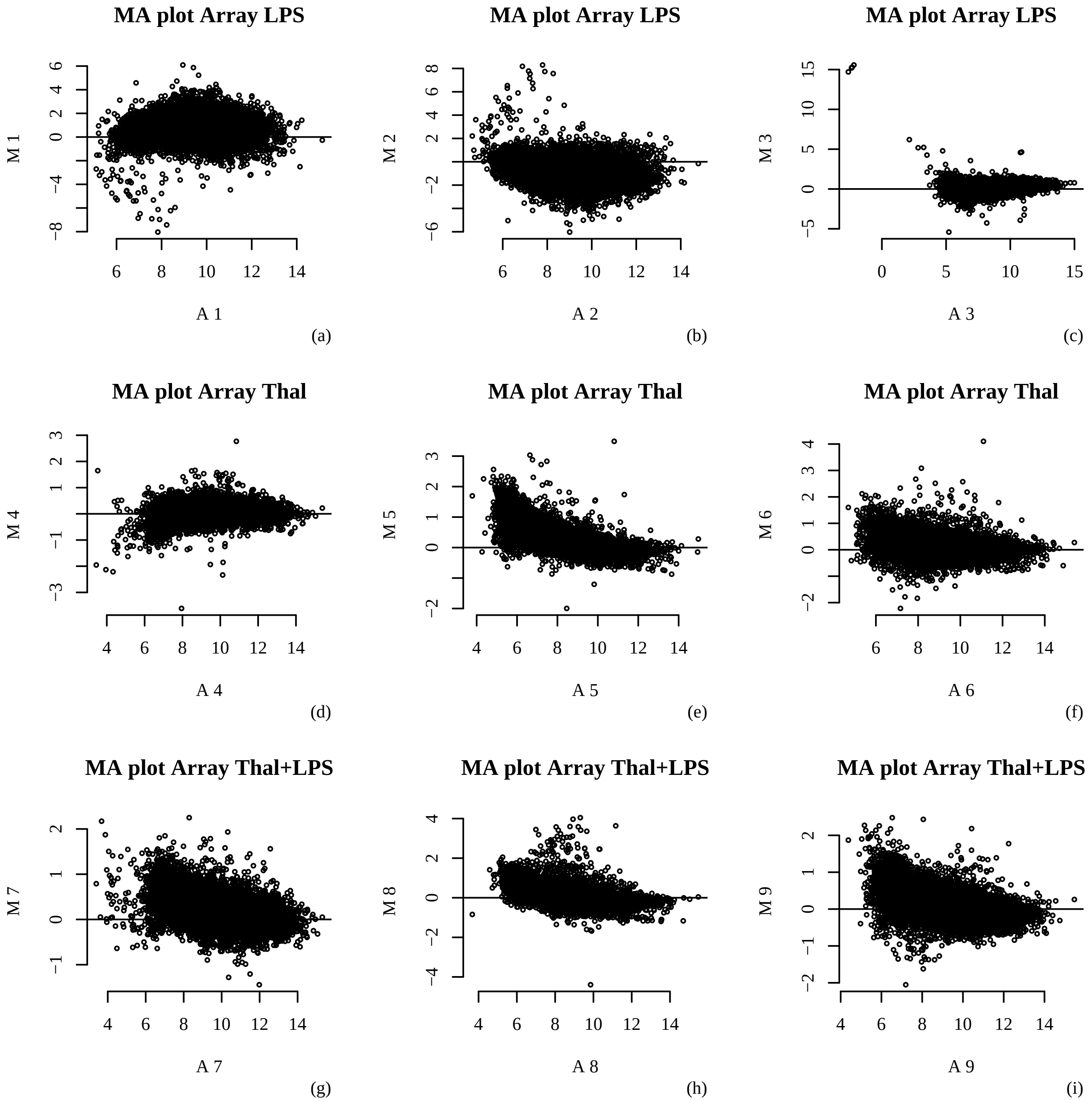
MA plot Array LPS
M 1
A 1
(a)
MA plot Array LPS
M 2
A 2
(b)
MA plot Array LPS
M 3
A 3
(c)
MA plot Array Thal
M 4
A 4
(d)
MA plot Array Thal
M 5
A 5
(e)
MA plot Array Thal
M 6
A 6
(f)
MA plot Array Thal+LPS
M 7
A 7
(g)
MA plot Array Thal+LPS
M 8
A 8
(h)
MA plot Array Thal+LPS
M 9
A 9
(i)
