# Supplementary material for: A framework to identify gene expression profiles in a model of inflammation induced by lipopolysaccharide after treatment with thalidomide
Source: BMC Res Notes. 2012 Jun 13;5:292. doi: 10.1186/1756-0500-5-292 (PMC3434117; doi:10.1186/1756-0500-5-292)
Supplement: Additional file 4: Table A.1_2 — Table A.1 Correlation between the arrays for the three experimental conditions and Table A.2 Correlation within arrays for the three experimental conditions. [file 1756-0500-5-292-S4.pdf]

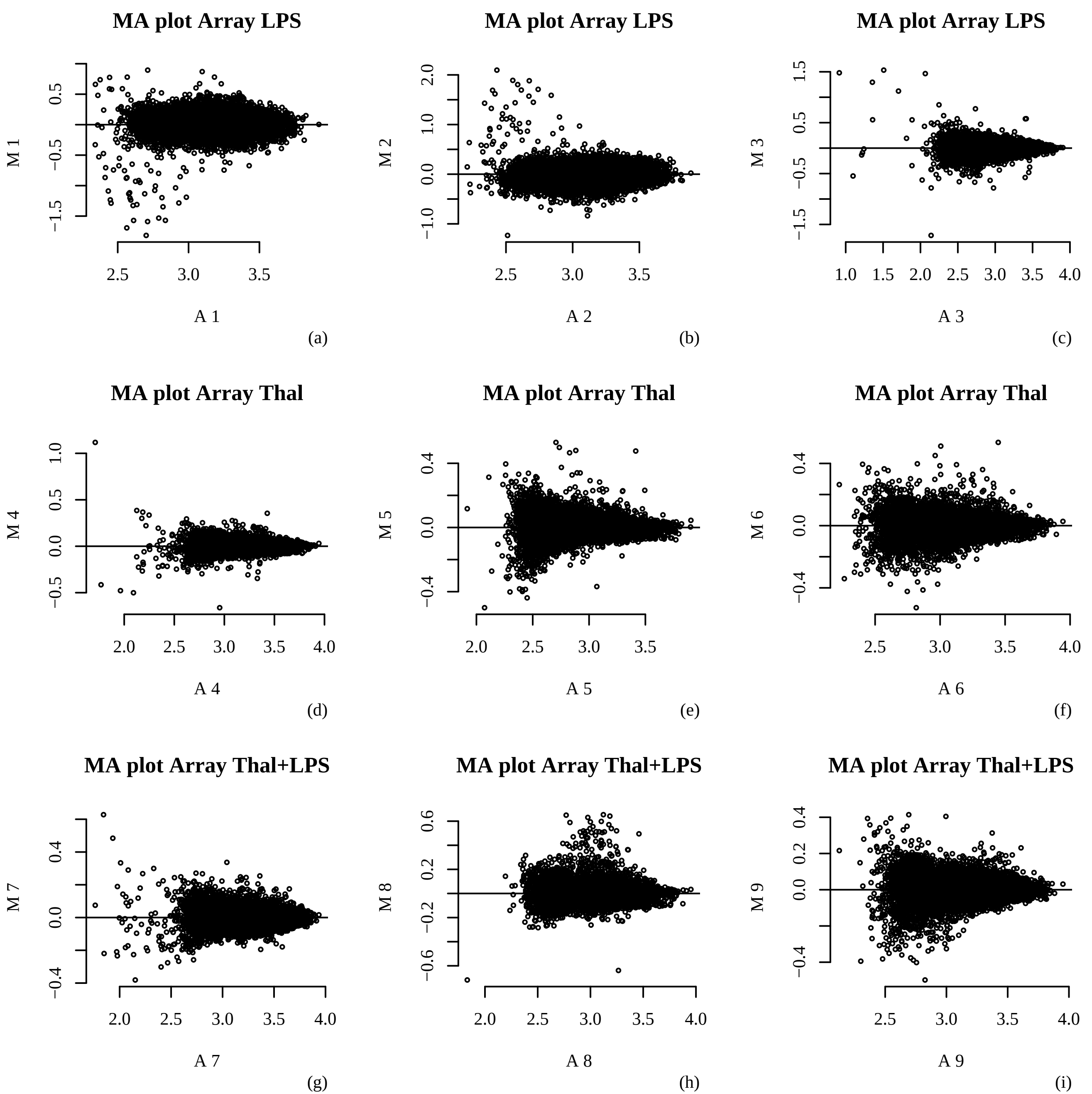
MA plot Array LPS
M 1
A 1
0.5
−0.5
−1.5
2.5
3.0
3.5
(a)
MA plot Array LPS
M 2
A 2
2.0
1.0
0.0
−1.0
2.5
3.0
3.5
(b)
MA plot Array LPS
M 3
A 3
1.5
0.5
−0.5
−1.5
1.0
1.5
2.0
2.5
3.0
3.5
4.0
(c)
MA plot Array Thal
M 4
A 4
1.0
0.5
0.0
−0.5
2.0
2.5
3.0
3.5
4.0
(d)
MA plot Array Thal
M 5
A 5
0.4
0.0
−0.4
2.0
2.5
3.0
3.5
(e)
MA plot Array Thal
M 6
A 6
0.4
0.0
−0.4
2.5
3.0
3.5
4.0
(f)
MA plot Array Thal+LPS
M 7
A 7
0.4
0.0
−0.4
2.0
2.5
3.0
3.5
4.0
(g)
MA plot Array Thal+LPS
M 8
A 8
0.6
0.2
−0.2
−0.6
2.0
2.5
3.0
3.5
4.0
(h)
MA plot Array Thal+LPS
M 9
A 9
0.4
0.2
0.0
−0.4
2.5
3.0
3.5
4.0
(i)
