# Supplementary figures and images for: A framework to identify gene expression profiles in a model of inflammation induced by lipopolysaccharide after treatment with thalidomide
Source: BMC Res Notes. 2012 Jun 13;5:292. doi: 10.1186/1756-0500-5-292 (PMC3434117; doi:10.1186/1756-0500-5-292)

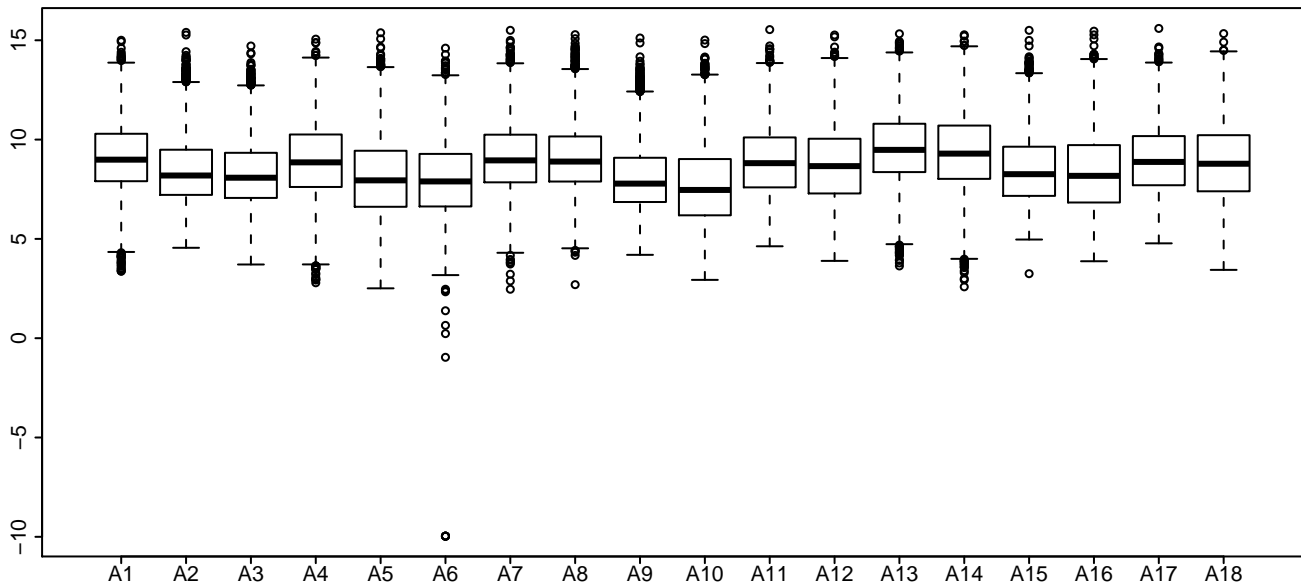

(a)

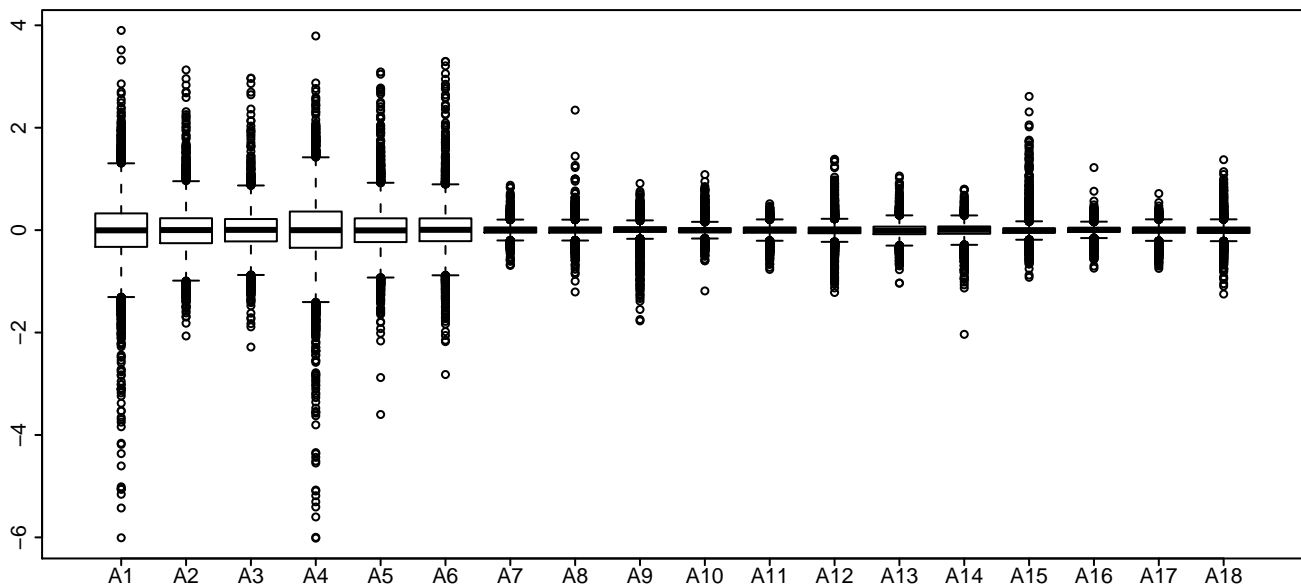

(b)

Supplement: Additional file 5: Table B1 — Genes identified as changed by LPS, as reported in this work, and those of SHARIF et al. [19] and LEE et al. [20]. [file 1756-0500-5-292-S5.pdf]
